# Aging Is Accompanied by a Blunted Muscle Protein Synthetic Response to Protein Ingestion

**DOI:** 10.1371/journal.pone.0140903

**Published:** 2015-11-04

**Authors:** Benjamin Toby Wall, Stefan H. Gorissen, Bart Pennings, René Koopman, Bart B. L. Groen, Lex B. Verdijk, Luc J. C. van Loon

**Affiliations:** NUTRIM School for Nutrition, Toxicology and Metabolism, Maastricht University Medical Centre, Maastricht, 6200 MD, The Netherlands; University of Birmingham, UNITED KINGDOM

## Abstract

**Purpose:**

Progressive loss of skeletal muscle mass with aging (sarcopenia) forms a global health concern. It has been suggested that an impaired capacity to increase muscle protein synthesis rates in response to protein intake is a key contributor to sarcopenia. We assessed whether differences in post-absorptive and/or post-prandial muscle protein synthesis rates exist between large cohorts of healthy young and older men.

**Procedures:**

We performed a cross-sectional, retrospective study comparing *in vivo* post-absorptive muscle protein synthesis rates determined with stable isotope methodologies between 34 healthy young (22±1 y) and 72 older (75±1 y) men, and post-prandial muscle protein synthesis rates between 35 healthy young (22±1 y) and 40 older (74±1 y) men.

**Findings:**

Post-absorptive muscle protein synthesis rates did not differ significantly between the young and older group. Post-prandial muscle protein synthesis rates were 16% lower in the older subjects when compared with the young. Muscle protein synthesis rates were >3 fold more responsive to dietary protein ingestion in the young. Irrespective of age, there was a strong negative correlation between post-absorptive muscle protein synthesis rates and the increase in muscle protein synthesis rate following protein ingestion.

**Conclusions:**

Aging is associated with the development of muscle anabolic inflexibility which represents a key physiological mechanism underpinning sarcopenia.

## Introduction

Sarcopenia refers to the progressive loss of skeletal muscle mass that occurs with normal, healthy aging [1]. Consequences of sarcopenia include an increased risk of falls/fractures, loss of independence and a greater likelihood of developing chronic metabolic diseases (e.g. type 2 diabetes). Given older individuals represent a growing proportion of our population [2], sarcopenia will have a profound impact on population health and associated healthcare systems in the years to come.

From a physiological standpoint, skeletal muscle mass is maintained by the parallel and opposing processes of muscle protein synthesis and breakdown. Relevant gains or losses of skeletal muscle mass are attributed to a persistent change in muscle protein synthesis rates, breakdown rates, or a combination of both. It is generally accepted that alterations in muscle protein synthesis play a more significant role in mediating long-term changes in muscle mass in healthy humans [3, 4]. Daily muscle protein synthesis rates are regulated in large part by the responsiveness to anabolic stimuli, such as food intake and physical activity [5]. Dietary protein and/or amino acid intake strongly increase muscle protein synthesis rates and inhibit muscle protein breakdown, thereby allowing net muscle protein accretion. These post-prandial periods offset the loss of muscle protein during post-absorptive conditions. Accordingly, both basal muscle protein turnover rates as well as the post-prandial stimulation of muscle protein synthesis are viewed as the key sites upon which skeletal muscle mass maintenance is regulated.

In an effort to explain the factors responsible for age-related muscle loss most research groups started to compare *in vivo* basal muscle protein synthesis rates between healthy young and elderly individuals. While some studies reported lower basal muscle protein synthesis rates in older men [6–10], other studies, often including larger cohorts (e.g. [11]), were unable to show any differences [11–18], whereas others actually reported higher muscle protein synthesis rates in the elderly [19]. As a consequence, many research groups have now switched their focus towards the muscle protein synthetic response to feeding. A few studies have shown that muscle protein synthesis rates are not increased to the same extent in senescent compared to younger muscle following either intravenous [17, 20] or oral [13, 15] essential/mixed amino acid administration, generally performed under euglycemic, hyperinsulinemic clamp conditions. This has led to the concept of anabolic resistance with aging [13, 15, 21]. However, other work from various laboratories has been unable to detect anabolic resistance in older populations in response to amino acid infusion [22–24], essential amino acid ingestion [16, 18] or the consumption of dietary protein [25–28] in more physiological (non-clamped) conditions.

We hypothesize that a reduced muscle protein synthetic response to feeding is evident in the older population. However, differences in post-prandial muscle protein synthesis rates between young and older individuals are difficult to detect in small stable isotope studies. Within our laboratory, over a ~5 year period, we have performed multiple stable isotope tracer studies to assess basal and/or post-prandial muscle protein synthesis rates in young and older men using the same study design. By collapsing these data we have the unique opportunity to compare basal muscle protein synthesis rates between 34 young and 72 elderly individuals, and post-prandial muscle protein synthesis rates between 35 young and 40 elderly subjects. This study is the first to provide evidence that aging is associated with an attenuated rise in muscle protein synthesis rate following dietary protein ingestion.

## Materials and Methods

### Data selection

The present paper is a retrospective, cross sectional study using data from six previously published studies from our laboratory [27, 29–33]. The studies were approved by the Medical Ethics Committee of the Maastricht University Medical Centre, Maastricht, the Netherlands in accordance with the Declaration of Helsinki. All subjects gave written informed consent prior to volunteering to take part in the study, and the records/information gathered were subsequently anonymized and de-identified prior to analysis. The included studies were selected as they all involved the use of a primed, constant *L*-[*ring*-^2^H_5_]phenylalanine stable isotope infusion with a minimum of 90 min pre-infusion time before data collection, followed by the determination of post-absorptive muscle protein synthesis rates assessed over a basal period of at least 2 h and the assessment of post-prandial muscle protein synthesis rates over a period of at least 4, and maximally 6, h. Post-prandial muscle protein synthesis data that were obtained over the first 2 h following protein ingestion were omitted from the present study as this is known to represent a peak muscle protein synthetic response [34], that is generally not representative of the integrated post-prandial muscle protein synthetic response to protein ingestion. For the comparisons of post-absorptive and post-prandial muscle protein synthesis rates, to minimize the heterogeneity in the dataset we only included data from studies that adhered to the following criteria: performed in healthy subjects (young and/or elderly); used *L*-[*ring*-^2^H_5_]phenylalanine constant intravenous infusions for the determination of muscle protein synthesis rates; and assessed the post-prandial response to the same amount and type of a single bolus of dietary protein (20 g casein). For the comparison of young and elderly subjects’ muscle protein synthesis rates, data obtained from experimental arms which also involved exercise [27], co-ingestion of other macronutrients [33], (following) an immobilization intervention [31] or ingestion of differing amounts [29] or types [32] of dietary protein, were omitted from the analysis. This approach yielded data from 34 young and 72 elderly men for the post-absorptive comparison, and 35 young and 40 elderly men for the post-prandial comparison and these subjects’ characteristics are presented in Table 1. To assess the delta change in muscle protein synthesis from fasted to fed, only those subjects were selected for whom post-absorptive and post-prandial muscle protein synthesis rates were assessed within the same experiment, and for these analyses we also included data with co-ingestion of carbohydrate (equivalent number between groups) allowing us *n* = 55 (22 young and 23 elderly). Further analysis was performed to assess the relationship between an individual’s post-absorptive muscle protein synthesis rate and the delta increase following protein ingestion. For these analyses, again only those subjects were selected for whom post-absorptive and post-prandial muscle protein synthesis rates were assessed within the same experiment, and for these analyses we also included subjects who had received different types/amounts of protein (*n* = 90; young = 22, elderly = 68).

**Table 1:** Subjects’ characteristics for comparisons of muscle protein synthesis rates.

	Post-absorptive			Post-prandial		
	*Young*	*Elderly*	*Significance*	*Young*	*Elderly*	*Significance*
***n***	34	72	---	35	40	---
**Age (y)**	22±1	75±1	*P*<0.001	22±1	74±1	*P*<0.001
**Lean body mass (kg)**	61.8±1.0	59.8±0.8	*P* = 0.12	61.0±1.2	56.6±1.1	*P*<0.05
**Body fat (%)**	16.8±0.8	21.1±0.4	*P*<0.001	16.7±0.8	20.3±0.6	*P*<0.001
**Leg lean mass (kg)**	10.8±0.2	9.2±0.1	*P*<0.001	10.6±0.3	8.9±0.2	*P*<0.001
**Glycated hemoglobin (%)**	5.4±0.1	5.7±0.1	*P*<0.001	5.4±0.1	6.0±0.1	*P*<0.001
**HOMA-IR index**	1.2±0.1	3.3±0.3	*P*<0.001	1.7±0.2	2.1±0.2	*P* = 0.21
**Fasting plasma insulin (mU** ^**.**^ **L** ^**-1**^ **)**	6±1	15±1	*P*<0.001	8±1	9±1	*P* = 0.20
**Fasting plasma leucine (μmol** ^**.**^ **L** ^**-1**^ **)**	129±3	130±2	*P* = 0.89	122±3	123±3	*P* = 0.87
**Peak post-prandial insulin (mU** ^**.**^ **L** ^**-1**^ **)**	---	---	---	18±2	20±2	*P* = 0.54
**Time to post-prandial peak insulin (m)**	---	---	---	32±4	31±2	*P* = 0.83
**Peak post-prandial leucine (μmol** ^**.**^ **L** ^**-1**^ **)**	---	---	---	235±7	273±9	*P*<0.01
**Time to post-prandial peak leucine (m)**	---	---	---	89±13	79±11	*P* = 0.55

Values are means±SEM. Abbreviations: HOMA-IR; Homeostatic Model Assessment of Insulin Resistance [57].

### Study characteristics

Specific screening details can be found in the individual studies [27, 29–33]. Additionally, all subjects received a similar standardized meal the evening prior to the stable isotope infusion experimental visits (33±2 kJ^.^kg^-1^ body weight, providing 44 energy% (En%) carbohydrate, 22 En% protein, and 34 En% fat). All volunteers were instructed to refrain from strenuous physical activity, avoid alcohol intake and to keep their diet as constant as possible for 2 days prior to the experimental test day. All experimental procedures were carried out at Maastricht University Medical School, the Netherlands.

### Calculations and analyses

The primary outcome measures for the present study were fractional rates of mixed muscle protein synthesis (FSR), calculated by dividing the increment in enrichment in the product (i.e. muscle protein-bound L-[*ring*-^2^H_5_]phenylalanine) by the enrichment of the precursor using the standard precursor-product relationship as previously described [32, 35]. For all data used in the present study, the plasma tracer enrichments (mean enrichment, weighted mean enrichment or incremental area under the curve) were selected as the precursor to calculate FSR. L-[*ring*-^2^H_5_]phenylalanine enrichments in plasma and mixed muscle protein were determined by GC-MS (Agilent 7890A GC/5975C; MSD, Little Falls, US) as previously described [32, 36].

For the purposes of subject classification and description, body composition was determined by dual energy x-ray absorptiometry (DEXA) scan (Hologic Inc., Bedford, USA) as previously described [37]. Blood samples for each individual were collected, processed and used to analyze plasma glucose, insulin and leucine concentrations, and blood HbA1c levels as described previously [29, 32].

### Statistics

All data are expressed as means±SEM. Differences between young and older subjects’ characteristics were each analyzed separately using unpaired *t*-tests. Post-absorptive and post-prandial muscle protein synthesis rates in young and older subjects were analyzed with a two-way ANOVA (age × prandial status) and, following a significant interaction effect, a Bonferroni post-hoc test was applied to locate individual differences. The delta increase in muscle protein synthesis rate with protein ingestion was compared between young and elderly subjects with an unpaired *t*-test using only data for whom post-absorptive and post-prandial muscle protein synthesis rates were assessed within the same experiment. For correlational analysis, a two-tailed Pearson’s Correlation Coefficient test was used. Correlational analysis was first performed with all data collapsed (i.e. young and elderly together) and subsequently for young and elderly cohorts separately. Statistical significance was set at *P*<0.05. All calculations were performed by using GraphPad Prism version 5.0 (GraphPad Software, San Diego, CA).

## Results

### Subjects’ characteristics

The subjects’ characteristics are displayed in Table 1. In general, younger subjects showed greater insulin sensitivity (reflected by lower glycated hemoglobin, fasting insulin and HOMA-IR values), lower body fat mass and a greater whole body and/or leg lean mass.

### Age-related comparisons of muscle protein synthesis rates

**Fig 1:**
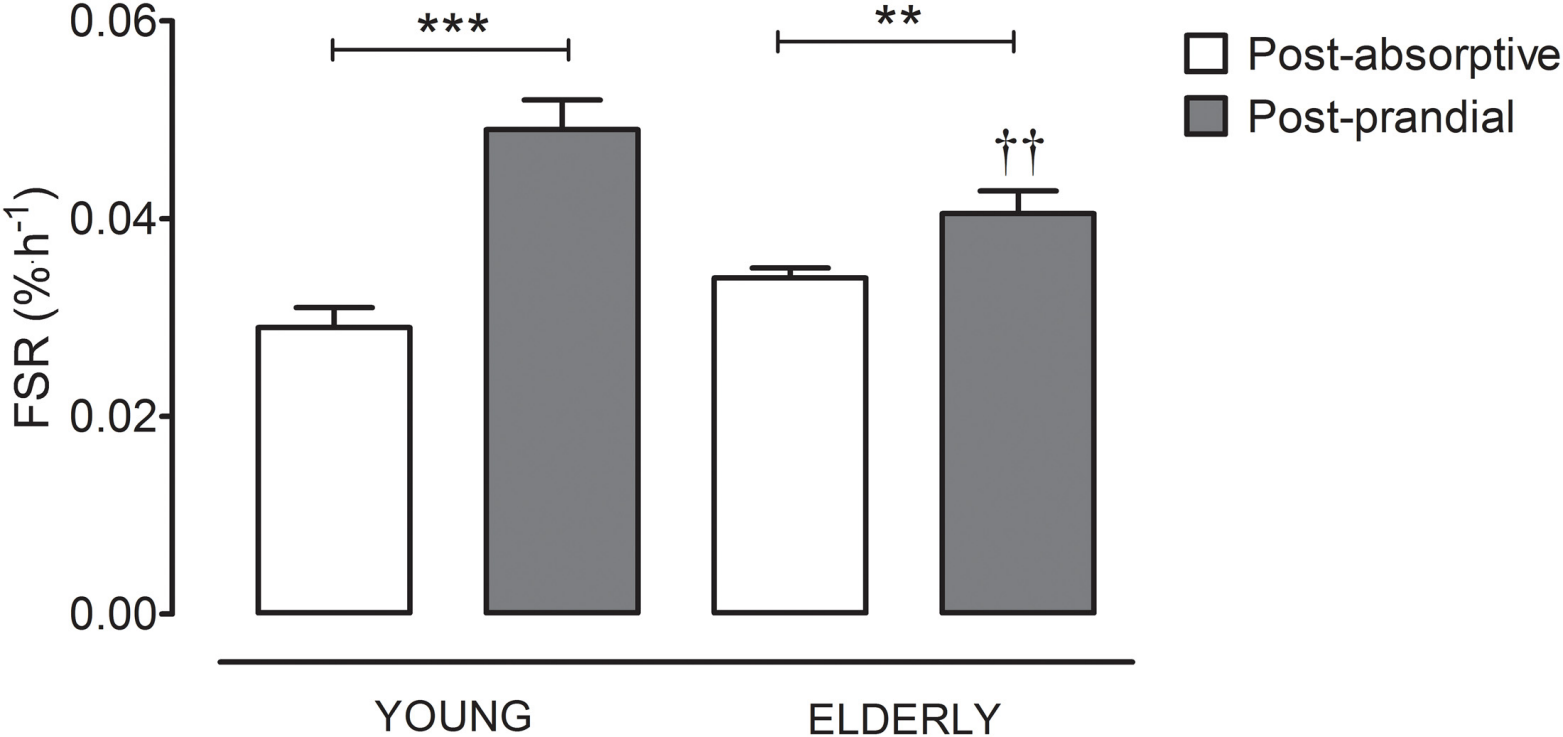
Fractional mixed muscle protein synthesis rates (FSR), calculated using plasma L-[*ring*-^2^H_5_] phenylalanine enrichments as the precursor pool, in healthy young and elderly men in the post-absorptive state (*n* = 34 young and *n* = 72 elderly) and following the ingestion of 20 g protein (post-prandial; *n* = 35 young and *n* = 40 elderly). Data were analyzed with multiple unpaired *t*-tests. Significantly different between corresponding post-absorptive and post-prandial values = ** (*P*<0.01), *** (*P*<0.001). Significantly different compared to corresponding values in the young = †† (P<0.01).

**Fig 2:**
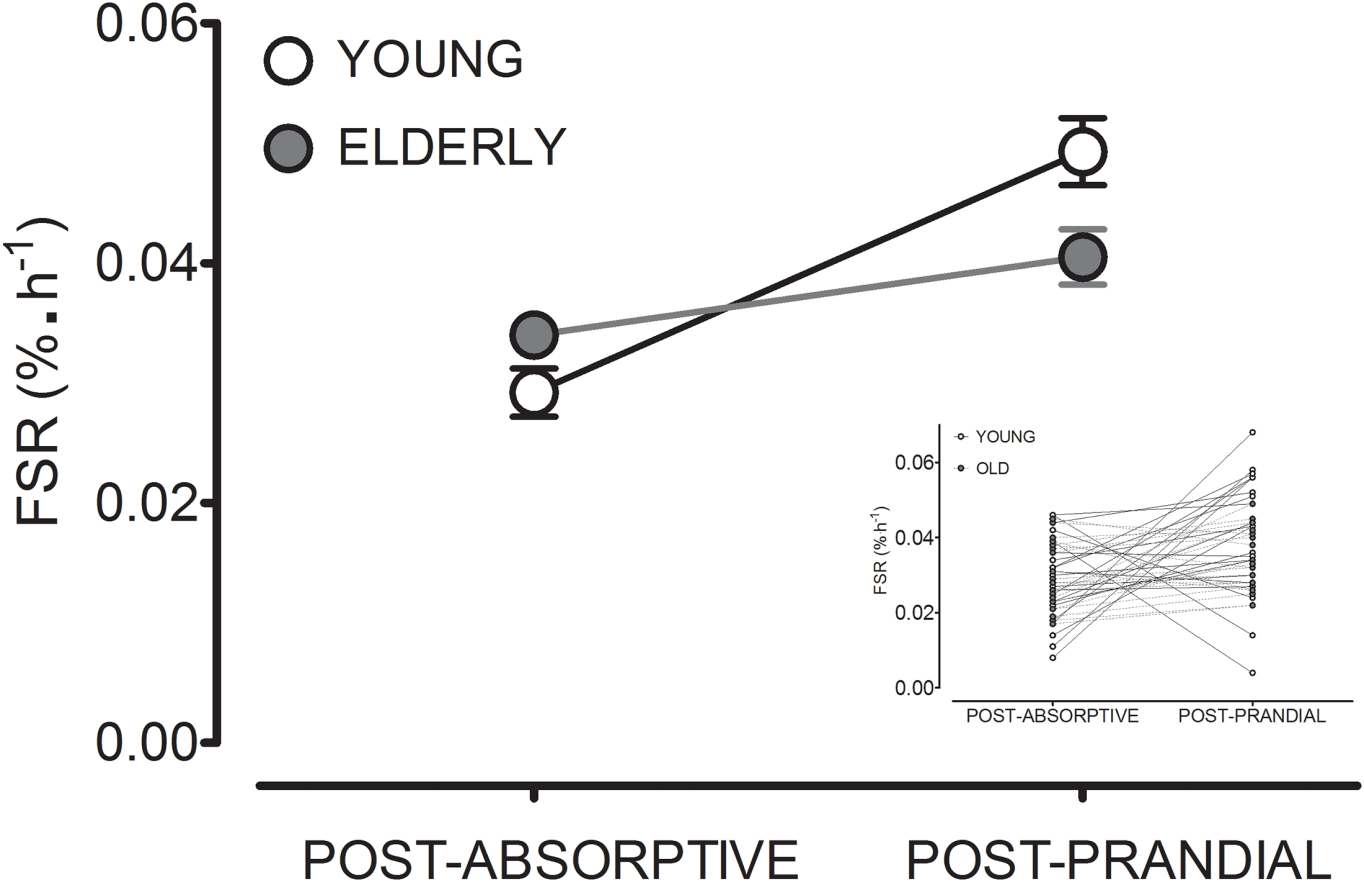
Fractional mixed muscle protein synthesis rates (FSR), calculated using plasma L-[*ring*-^2^H_5_] phenylalanine enrichments as the precursor pool, in healthy young and elderly men in the post-absorptive state (*n* = 34 young and *n* = 72 elderly) and following the ingestion of 20 g protein (post-prandial; *n* = 35 young and *n* = 40 elderly. Delta increases from post-absorptive to post-prandial were analyzed between groups using an unpaired *t*-test (significantly different; *P*<0.01). Individual responses are reported in the inset figure only in those subjects for whom post-absorptive and post-prandial muscle protein synthesis rates were assessed within the same experiment (*n* = 55 [22 young and 23 elderly]).

Mean post-absorptive and post-prandial muscle protein synthesis rates for young and older men are presented in Fig 1. Post-absorptive muscle protein synthesis rates did not differ between young and older men (0.028±0.002 vs 0.034±0.001%^.^h^-1^, respectively: significant interaction; *P*<0.001, but post-absorptive rates not different with Bonferroni; *P*>0.05). Post-prandial muscle protein synthesis rates (0.049±0.003 and 0.041±0.002%^.^h^-1^ for young and elderly subjects, respectively) were significantly greater than post-absorptive rates in both age groups, by 75 and 21% for young and older men, respectively (significant main effect of prandial status; *P*<0.0001). Post-prandial muscle protein synthesis rates were 16% lower in older men when compared with the young (significant interaction; *P*<0.001, post-prandial rates different with Bonferroni; *P*<0.01). The delta increase in muscle protein synthesis rates from the post-absorptive to post-prandial state was substantially greater in the young compared with the older men (*P*<0.01; Fig 2).

**Fig 3:**
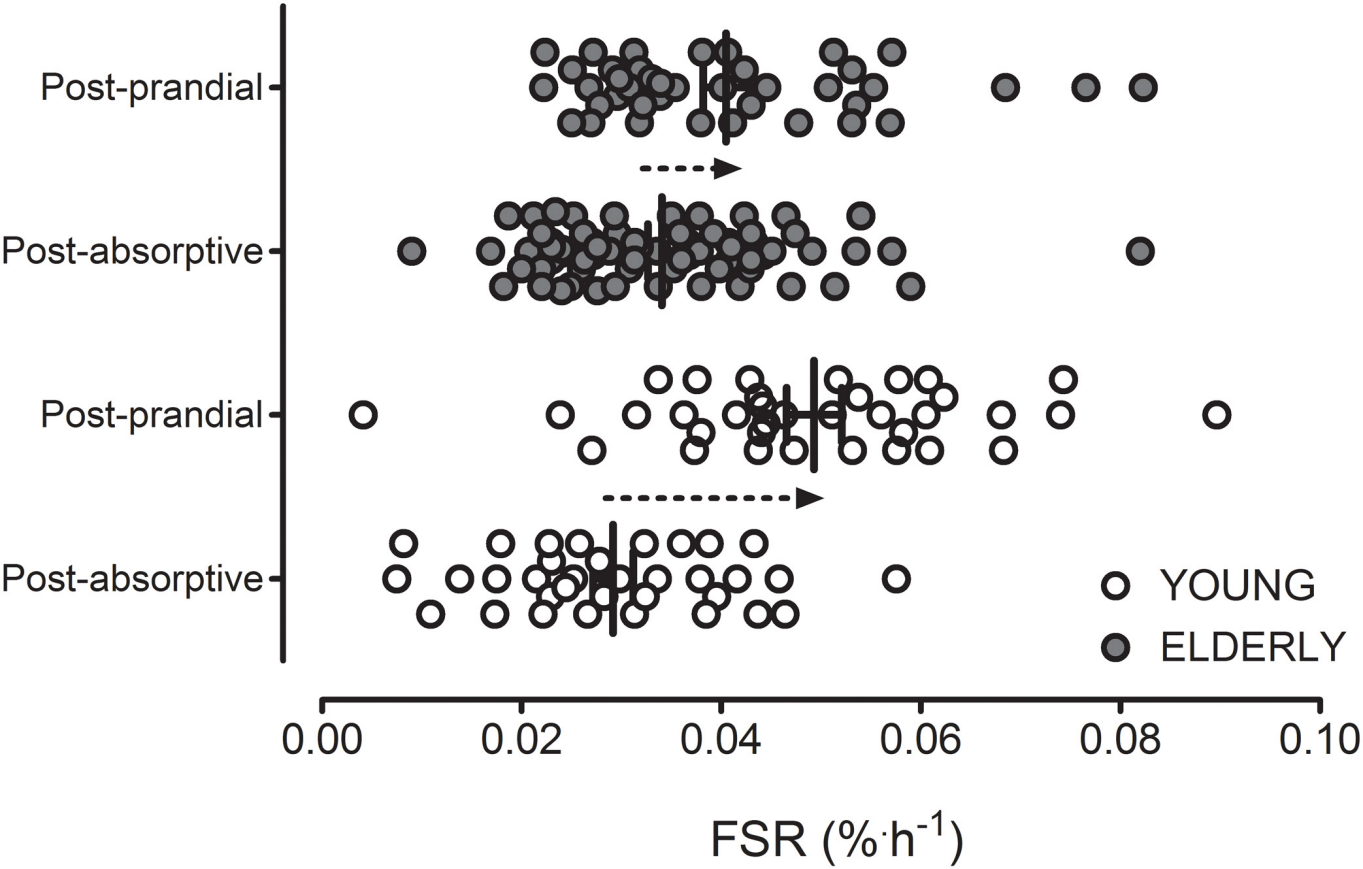
Graphical representation of the individual values for fractional mixed muscle protein synthesis rates (FSR), calculated using plasma L-[*ring*-^2^H_5_]phenylalanine enrichments as the precursor pool, in healthy young and elderly men in the post-absorptive state (*n* = 34 young and *n* = 72 elderly) and following the ingestion of 20 g protein (post-prandial; *n* = 35 young and *n* = 40 elderly).

**Fig 4:**
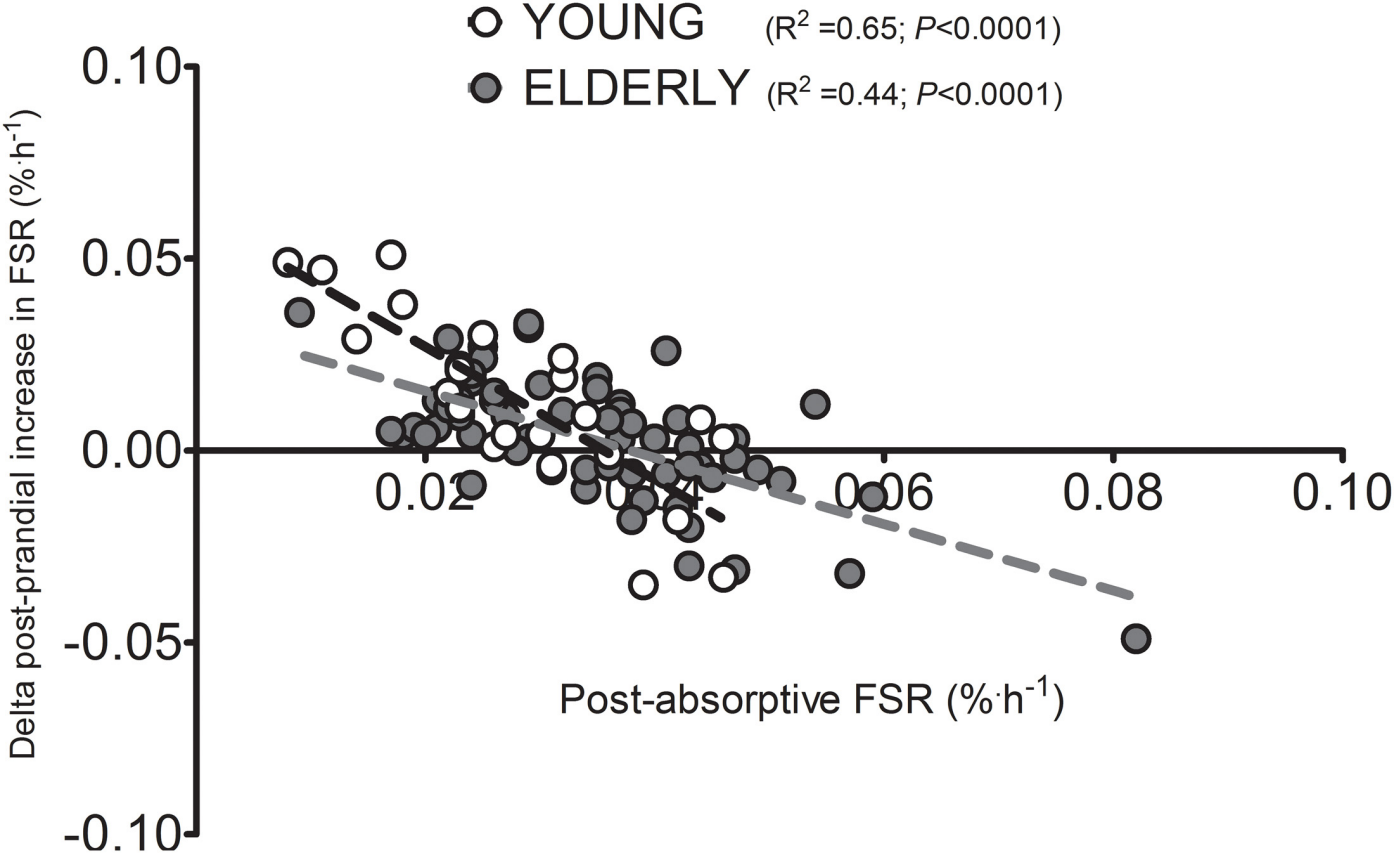
Correlation between post-absorptive fractional mixed muscle protein synthesis rates (FSR) and the delta increase in FSR following the ingestion dietary protein (post-prandial) in healthy young (*n* = 22) and elderly (*n* = 68) men. Data were analyzed with 2-tailed tests of significance by using Pearson's correlation coefficients.

Interestingly, if it is assumed that 12 h of each day is spent under post-absorptive and 12 h under post-prandial conditions, the amount of muscle tissue synthesized per day taking account of lean body mass of the subjects can be calculated. In this regard, an equivalent amount of daily muscle protein is synthesized under post-absorptive conditions per day between young and older subjects (21±2 and 24±2 g, respectively; *P*>0.05). However, under post-prandial conditions, younger subjects synthesized considerably more than the elderly (36±2 and 28±2 g, respectively; *P*<0.001). When post-absorptive and post-prandial muscle protein synthesis rates for young and older men were expressed per kg lean body mass, no differences in post-absorptive muscle protein synthesis rates were observed but older subjects displayed 30% lower post-prandial muscle protein synthesis rates compared with the young (*P*<0.01). Moreover, when adjusted for lean body mass, only the young cohort demonstrated a significantly greater (65%) muscle protein synthetic response to protein feeding when compared with basal (*P*<0.001). Fig 3 illustrates the individual values of post-absorptive and post-prandial muscle protein synthesis rates for young and older men. In addition, we examined the relationship between post-absorptive muscle protein synthesis rates and the magnitude of increase following protein ingestion where both measurements had been made in an individual during a single visit (Fig 4). Regardless of age, a strong negative correlation (*r* = -0.70; *P*<0.001) was observed between basal muscle protein synthesis rates and the magnitude of stimulation following protein ingestion. Similar correlations were observed within the young and elderly sub-groups separately (*r* = -0.81 and *r* = -0.66, respectively; both *P*<0.0001). These correlations were also evident when muscle protein synthesis rates adjusted for lean body mass were used.

## Discussion

The present study demonstrates that an advanced age is associated with a blunted muscle protein synthetic response to protein ingestion and, irrespective of age, a strong negative relationship was shown to exist between basal muscle protein synthesis rates and the anabolic response to protein ingestion.

It has been proposed that a decline in basal (post-absorptive) muscle protein synthesis rates could explain age-related muscle loss. In support, several studies have reported that older men express lower basal muscle protein synthesis rates [6–10]. However, various other investigations failed to confirm these findings [11–19] which included the largest cohort studied to date (prior to this study) [11]. The lack of age-related differences in these studies may be attributed to the limited number of subjects that are generally included in these expensive stable isotope infusion studies. By collating data from multiple studies performed within our laboratory over the past 5 years [27, 29–33], we were able to assess basal muscle protein synthesis rates in a large number (>100) of healthy young and older men using the exact same study design. We report no statistical difference (and actually numerically *higher* values in the elderly) in basal muscle protein synthesis rates at a more advanced age (Fig 1). Evidently, a decline in post-absorptive muscle protein synthesis rates does not provide a valid explanation for the loss of muscle mass observed with aging.

Food ingestion strongly increases muscle protein synthesis rates beyond basal levels for a period of up to 3–5 hours, allowing net muscle protein accretion [5]. The post-prandial increase in muscle protein synthesis rate is, therefore, believed to represent a key regulatory factor allowing muscle mass maintenance in healthy individuals [5]. Recent work suggests that this anabolic response to feeding may be compromised at a more advanced age. Several studies have reported a blunted muscle protein synthetic response to intravenous [17, 20] or oral [13, 15] essential/mixed amino acid administration in older adults, generally performed under insulin clamped conditions. These data have resulted in the hypothesis that the loss of muscle mass with aging is, at least partly, attributed to anabolic resistance to food intake [13, 15, 21]. Follow-up studies have attempted to test this hypothesis by measuring the post-prandial muscle protein synthetic response to the ingestion of amino acids or meal-like amounts of dietary protein. However, these studies have been unable to confirm the presence of anabolic resistance in older individuals [16, 18, 22–28]. It is possible that these studies have failed to capture small, clinically relevant differences in the post-prandial muscle protein synthetic response to protein ingestion between young and older subjects simply due to the small numbers of subjects necessarily included in these studies. The extensive data set presented here (Fig 1) shows that the 4–6 h post-prandial muscle protein synthetic response to the ingestion of dietary protein is substantially blunted in older men when compared with younger controls. The same absolute amount (20 g) and type (casein) of dietary protein was consumed by both the young and older subjects, irrespective of the lower quantity of lean mass in the older subjects (Table 1). Despite the greater absolute amount of protein consumed per kg lean body mass in the older group (also resulting in higher levels of circulating leucine; see Table 1), we still observe an attenuated post-prandial muscle protein synthetic response to protein ingestion in the older compared with younger males. It is important to note that, even when data are adjusted for lean body mass when comparing the two populations, anabolic resistance was still clearly evident. This is the first study to confirm the presence of anabolic resistance in response to dietary protein ingestion in healthy older adults, implying that anabolic resistance to normal food consumption is a key factor responsible for age related muscle loss.

Although post-prandial muscle protein synthesis rates were substantially lower in the older compared with the younger men, an even more striking difference between groups was the reduced capacity to elevate basal muscle protein synthesis rates following the ingestion of a given quantity of dietary protein. The older men showed a more than 3-fold smaller capability to elevate muscle protein synthesis rate in response to feeding when compared with the younger males (Fig 2). Interestingly, this reduced sensitivity to respond to the anabolic properties of dietary protein bears a striking resemblance to the concept of metabolic inflexibility that heralds the development of insulin resistance and chronic metabolic disease [38–40]. Metabolic inflexibility traditionally refers to a reduced ability of skeletal muscle tissue to adjust fuel selection to alterations in substrate availability [38–40]. In a similar fashion, the present data indicate that senescent muscle is less capable of facilitating an appropriate increase in muscle protein synthesis rate in response to a rise in plasma amino acid availability. As such, we introduce the concept of anabolic inflexibility with aging. Indeed it is also apparent that, on an individual basis, the basal level of muscle protein synthesis is intrinsically linked with the capacity for its stimulation following food ingestion (Fig 4). This highlights the importance of assessing the sensitivity by which *basal* muscle protein synthesis rates are increased following the ingestion of a given dose of protein, and not only the absolute post-prandial muscle protein synthetic rate. It is apparent that similar post-prandial muscle protein synthesis rates can actually be achieved in young and older men, though the elderly require higher doses of dietary protein to reach equivalent muscle protein synthesis rates [41]. Consequently, the reduced capacity to mount an appropriate rise in muscle protein synthesis rates following ingestion of smaller, meal-like quantities of protein may represent the specific key impairment in muscle protein metabolism that occurs at a more advanced age.

The present data illustrate the development of anabolic resistance and inflexibility with aging. Whether anabolic inflexibility can be attributed to aging *per se* is a key question that needs to be addressed. We recently argued that age-related anabolic resistance may be largely attributed to lower physical activity levels in the older population [21, 42]. In support, we and others have demonstrated that physical inactivity/muscle disuse leads to the rapid development of anabolic resistance in both young [31, 43] and older [44, 45] subjects. However, it is also true that reduced physical activity could also reasonably be expected to lower basal muscle protein synthesis rates [43, 46, 47], which was not the case in the present study (assuming lower physical inactivity levels in the elderly). It is possible that the anabolic response to protein ingestion is more susceptible (compared with basal protein metabolism) to changes associated with physical activity status, as observed by Drummond and colleagues [44]. This would suggest that anabolic responsiveness, rather than basal muscle protein metabolism, may exert a greater influence on long term muscle mass maintenance, though this assertion requires further investigation. Similarly, nutritional factors may impact upon the degree of anabolic flexibility with aging. For example, reduced energy intake lowers muscle protein synthesis rates [48]. Though acute (3 d) manipulations of dietary protein intake do not appear to alter muscle protein metabolism [49], little is known in terms of how longer term *habitual* protein consumption may modulate acute anabolic responses to protein containing meals. Several other biological, lifestyle or environmental factors, such as smoking [50], alcohol consumption [51], body composition [52], hormonal status [53], and the presence of chronic metabolic disease [52, 54–56], tend to change with increasing age and may strongly influence muscle protein metabolism. These factors may also explain the large variance in basal and post-prandial muscle protein synthesis rates that were observed between subjects (Fig 3). In addition, it should be acknowledged that, in real life, protein is usually consumed as part of a mixed meal, which depending upon its composition may have the capacity to alter the anabolic properties of the ingested protein. So far, however, no studies have comprehensively evaluated muscle protein synthesis rates in response to the consumption of mixed meals. This study is the first to collect an extensive data set to evaluate both the basal and post-prandial muscle protein synthesis rates following ingestion of a meal like amount of intact dietary protein. Collectively, the identification of factors that modulate anabolic flexibility will allow the development of more effective nutritional, exercise and/or pharmacological strategies to attenuate or prevent skeletal muscle loss with aging and/or disease.

The present study is the first to demonstrate that healthy older men experience an attenuated rise in muscle protein synthesis rate following the ingestion of a meal-like amount of protein. We conclude that the development of anabolic inflexibility contributes to the loss of muscle mass with aging.

## Supporting Information

S1 Data Raw data underlying main findings of the study. (XLS)

## Abbreviations

FSR: fractional synthetic rate
